# FOSTERING COMPASSIONATE CARE FOR PERSONS WITH ALZHEIMER’S DISEASE AND RELATED DEMENTIAS

**DOI:** 10.1093/geroni/igz038.1743

**Published:** 2019-11-08

**Authors:** Samantha G Cotton, Anna Faul, Joe D’Ambrosio, and Pamela Yankeelov

**Affiliations:** University of Louisville, Louisville, Kentucky, United States

## Abstract

The aim of this study was to examine the impact of the implementation of a new Compassionate Care (CC) curriculum, designed by social workers, on the quality of care provided by Certified Nursing Assistants (CNAs) to residents with Alzheimer’s disease (AD). Additionally, the purpose was to create a collaborative network of CNAs that supported each other. The sample included residents and CNAs from an experimental nursing facility and a control nursing facility. At baseline and 12-weeks, data were collected on AD knowledge, self-efficacy, caregiving satisfaction, and affiliate stigma. CNA changes in terms of their knowledge of AD, self-efficacy, caregiving satisfaction and affiliate stigma were analyzed using a two-way mixed method MANOVA. The stress levels of the residents, specifically agitation and salivary cortisol levels, was examined by testing a hybrid multilevel growth model. The final models were able to show how the changes in the CNAs specifically affected these positive outcomes. CNA knowledge and self-efficacy had the most impact on changing agitation levels, and CNA knowledge and agitation levels had the most impact on salivary cortisol levels. The results of this study showed that integrating a compassionate care curriculum into the work that CNAs perform can lead to positive outcomes on knowledge, self-efficacy, caregiving satisfaction, affiliate stigma and a reduction of agitation and cortisol levels in persons with AD.

